# Dynamics of serotype 14 *Streptococcus pneumoniae* population causing acute respiratory infections among children in China (1997–2012)

**DOI:** 10.1186/s12879-015-1008-7

**Published:** 2015-07-11

**Authors:** Mingming He, Kaihu Yao, Wei Shi, Wei Gao, Lin Yuan, Sangjie Yu, Yonghong Yang

**Affiliations:** Key Laboratory of Major Diseases in Children and National Key Discipline of Pediatrics, Ministry of Education, Beijing Pediatric Research Institute, Beijing Children’s Hospital Affiliated to Capital Medical University, Beijing, P. R. China

**Keywords:** *Streptococcus pneumoniae*, Serotypes, Antibiotic resistance, Children, Epidemiology

## Abstract

**Background:**

In the last decade, the *Streptococcus pneumoniae* population has changed, mainly due to the abuse of antibiotics. The aim of this study was to determine the genetic structure of 144 *S. pneumonia* serotype 14 isolates collected from children with acute respiratory infections during 1997–2012 in China.

**Methods:**

All isolated pneumococci were tested for their sensitivity to 11 kinds of antibiotics with the E-test method or disc diffusion. The macrolides resistance genes *ermB* and *mefA*, as well as the sulfamethoxazole-trimethoprim resistance gene dihydrofolate reductase (DHFR) were detected by polymerase chain reaction (PCR). The sequence types (STs) were analyzed with multilocus sequence typing (MLST).

**Results:**

From 1997 to 2012, the percentage of serotype 14 *S. pneumonia* isolates in the whole isolates increased. All of the 144 serotype 14 *S. pneumonia* isolates were susceptible to amoxicillin-clavulanic acid, vancomycin and levofloxacin. No penicillin resistant isolate was found, and the intermediate rate was as low as 0.7 %. Erythromycin resistance was confirmed among 143 isolates. The *ermB* gene was determined in all erythromycin resistant isolates, and the *mefA* gene was positive additionally in 13 of them. The non-susceptibility rate to the tested cephalosporins increased from 1997–2012. All trimethoprim-resistant isolates contained the Ile100-Leu mutation. Overall, 30 STs were identified, among which ST876 was the most prevalent, followed by ST875. During the study period, the percentage of CC876 increased from 0 % in 1997–2000 to 96.4 % in 2010–2012, whereas CC875 decreased from 84.2 to 0 %. CC876 showed higher non-susceptibility rates to β-lactam antibiotics than CC875.

**Conclusion:**

The percentage of serotype 14 *S. pneumonia* isolates increased over time in China. The increase of resistance to β-lactam antibiotics in this serotype isolates was associated with the spread of CC876.

## Background

*Streptococcus pneumoniae* (*S. pneumonia*) is a leading cause of bacterial infections in infants and young children. World Health Organization (WHO) estimated that 1.6 million people die of pneumococcal diseases annually, including 0.7–1 million children aged <5 years mostly from developing countries [1]. Management of *S. pneumonia* infections has been complicated by the emergence of multiple antimicrobial drug-resistant strains [2].

More than 92 serotypes have been identified based on the discrepancy of capsular polysaccharide antigens. Before the introduction of the pneumococcal conjugate vaccines, serotype 14 *S. pneumonia* was one of the most common types worldwide [3, 4] and it was usually one common type of antibiotic resistant isolates [5]. Penicillin-resistant and multidrug-resistant pneumococci were known to be restricted to serogroups 23, 6, 19 and serotype 14, which were particularly associated with the infectious diseases in children [6–8]. Spain^14–5^(ST18), England^14–9^(ST9), CSR^14–10^(ST20), Tennessee^14–18^(ST67), Denmark^14–32^(ST230) and Netherlands^14–35^(ST124) were internationally disseminated antimicrobial resistant clones described by the Pneumococcal Molecular Epidemiology Network (PMEN) (http://www.sph.emory.edu/PMEN/index.html).

We reported previously some international spread drug resistant clones in serotype 19 F and 23 F which had been identified in China with increasing frequency [9, 10]. The current study is conducted to analyze the antimicrobial resistance and population biology of serotype 14 *S. pneumonia* isolates in China.

## Methods

### Pneumococcal isolates

From 1997 to 2012, a total of 1984 *S. pneumonia* isolates were collected from children <5 years old with the diagnosis of acute upper respiratory infection in Beijing, Shanghai, Guangzhou, Shenzhen, Chongqing, Xi’an of China. The patients suffering from “lower respiratory tract infections” like pneumonia were excluded because we can not ensure the isolates were from lower respiratory tract or from the nasopharynx. The details of these S. pneumoniae collections have been published previously [11–16]. A total of 144 isolates were identified as serotype 14 based on quellung reaction, which was performed using the Pneumotest Kit (Statens Serum Institut, Copenhagen, Denmark). The isolates were cultured from nasopharyngeal specimens, except those collected from Beijing in 2006–2008 and from Shenzhen in 2009–2011, which were cultured from hypopharyngeal aspirate specimens.

All isolates were taken as part of standard patient care. A parent and/or legal guardian of each participant signed a written informed consent document before enrollment and before any study procedure was performed. This study was viewed and approved by the Ethics Committee of Beijing Children’s Hospital Affiliated to Capital Medical University. No ethical problems existed in this study.

### Antimicrobial susceptibility

The minimum inhibitory concentrations (MICs) of all isolates were determined for penicillin, amoxicillin–clavulanic acid, ceftriaxone, cefuroxime, erythromycin, imipenem, levofloxacin, and vancomycin using E-test strips (AB Biodisk, Solna, Sweden) [17], and their susceptibility to tetracycline, sulfamethoxazole–trimethoprim, and chloramphenicol was assessed with disc diffusion tests (Oxoid). The breakpoints were adopted in accordance with the Clinical and Laboratory Standards Institute 2012 criteria [18]. *Streptococcus pneumoniae* ATCC49619 was used as the reference strain in the susceptibility tests. Isolates were considered multidrug resistant if they were not susceptible to three or more classes of antimicrobials.

### Macrolide-resistance genes

Chromosomal DNA was extracted from overnight cultures of *S. pneumonia* isolates grown on 5 % trypticase soy agar (Oxoid Ltd, Basingstoke, England) using the SiMax™ Genomic DNA Extraction Kit (SBS Genetech Co., Ltd), according to the manufacturer’s instructions. The macrolide-resistance genes *ermB* and *mefA* were detected by polymerase chain reaction (PCR) for all of the erythromycin non-susceptible strains, the primers and PCR procedures were previously described [19].

### Multilocus sequence typing (MLST)

All strains were characterized with multilocus sequence typing (MLST). Bacterial chromosomal DNA was extracted as described above. The PCR fragments of the seven MLST genes (*aroE*, *gdh*, *gki*, *recP*, *spi*, *xpt*, and *ddl*) were amplified from the chromosomal DNA. The products were sent to BGI Company (Beijing, China) for sequencing on both strands. The STs were determined by comparing the allelic profiles with the recognized STs at the MLST website (http://spneumoniae.mlst.net). The new alleles and allelic profiles identified in the present study have been submitted to the MLST database for name assignment. The eBURST v3 software (available at http://www.mlst.net) was used to estimate the relationships among the isolates and to assign strains to a clonal complex (CC) using the stringent group definition of six of seven shared alleles.

### Dihydrofolate reductase genes

The dihydrofolate reductase (DHFR) genes were amplified for all isolates with primers previously reported [20] and then sequenced. The sequences were analyzed and compared to each other by MEGA4.1 software.

### Statistical analysis

The antibiotic susceptibility data were analyzed using WHONET 5.6 software as recommended by the WHO. The χ^2^ test, performed with the SPSS software v. 13.0 (SPSS Inc. USA), was used for statistical comparisons. For effective comparison, the study period was divided into five stages: 1997–2000, 2001–2003, 2004–2006, 2007–2009 and 2010–2012. A two-tailed cut-off of *P* < 0.05 indicated statistical significance.

## Results

### Frequency of serotype 14 *S. pneumonia* isolates over time

During the study period, serotype 14 isolates was identified in 7.3 % (144/1984) of all of the *S. pneumonia* strains. The frequencies in different stages were 3.2 % (19/593) in 1997–2000, 5.5 % (38/691) in 2001–2003, 7.1 % (24/338) in 2004–2006, 22 % (35/159) in 2007–2009, 13.8 % (28/203) in 2010–2012 (χ^2^ = 82.069, *P* < 0.05).

### Antibiotic susceptibility

The susceptibility and the MICs of the isolates against 11 antibiotics were presented in Table 1. All of the 144 strains were susceptible to amoxicillin-clavulanic acid, vancomycin and levofloxacin. Only one isolate was intermediate to penicillion (MIC: 3 μg/mL). The non-susceptible rates to cefuroxime, ceftriaxone, imipenem and tetracycline were 51.4 %, 8.3 %, 49.3 % and 85.4 %, respectively. A total of 143 (99.3 %) isolates were resistant to erythromycin with high MICs (139 isolates had a MIC ≥ 256 μg/mL). The resistance rates to trimethoprim-sulfamethoxazole and chloramphenicol were 47.2 % and 15.3 %, respectively. About 52.1 % (75/144) of all pneumococcal isolates were MDRSP, most of which were co-resistance to erythromycin, tetracycline, and sulfamethoxazole-trimethoprim.

**Table 1:** Susceptibility and MICs of 144 pneumococcal isolates to 11 antibiotics

Antibiotics	Susceptibility		MIC(μg/ml)		
	I(%)	R(%)	50 %	90 %	Range
PEN	0.7	0	0.5	1	0.004–3
AMC	0	0	0.38	1	0.008–2
CXM	43.8	7.6	1.5	3	0.008–6
CRO	6.9	1.4	0.38	1	0.004–8
IPM	49.3	0	0.094	0.19	0.004–0.38
ERY	0	99.3	>256	>256	0.125–> 256
LVX	0	0	0.5	0.75	0.25–1.5
VAN	0	0	0.5	0.75	0.094–1
TCY	28.5	56.9	-	-	-
SXT	0	47.2	-	-	-
CHL	0	15.3	-	-	-

* PEN* penicillin, *AMC* amoxicillin–clavulanic acid, *CXM* cefuroxime, *CRO* ceftriaxone, *IPM* imipenem, *ERY* erythromycin, *LVX* levofloxacin, *VAN* vancomycin, *TCY* tetracycline, *CHL* chloramphenicol, *SXT* trimethoprim–sulfamethoxazole, - no data for disk diffusion test, *I* intermediate, *R* resistant, *MIC50* minimum inhibitory concentration at which 50 % of the strains were inhibited, *MIC90* minimum inhibitory concentration at which 90 % of the strains were inhibited, *MIC range* range of minimum inhibitory concentration

The resistance rate of serotype 14 *S. pneumonia* isolates against β-lactam antibiotics showed an increasing trend (Table 2). From 1997 to 2012, the non-susceptibility rates to ceftriaxone, cefuroxime and imipenem increased. By contrast, the non-susceptibility rate to sulfamethoxazole-trimethoprim and chloramphenicol decreased from 84.2 % in 1997–2000 to 3.6 % in 2010–2012 and from 52.6 to 0 % in 2007–2009 and 2010–2012, respectively.

**Table 2:** Susceptibility of the 144 serotype 14 isolates to eight antimicrobials throughout the study period

Antibiotics	Susceptibility and MIC	1997–2000 (*n* = 19)	2001–2003 (*n* = 38)	2004–2006 (*n* = 24)	2007–2009 (*n* = 35)	2010–2012 (*n* = 28)	Total (*n* = 144)
PEN	I%	0	0	0	2.9	0	0.7
	MIC50(μg/ml)	0.016	0.032	0.5	0.75	0.5	0.5
	MIC90(μg/ml)	0.75	0.5	1	1.5	1	1
	MIC Range(μg/ml)	0.004–1.5	0.008–0.75	0.012–1.5	0.008–3	0.25–1.5	0.004–3
AMC	MIC50(μg/ml)	0.016	0.023	0.5	0.5	0.5	0.38
	MIC90(μg/ml)	0.75	0.5	1	1	0.75	1
	MIC Range(μg/ml)	0.008–1	0.008–1	0.016–2	0.008–1	0.25–1.5	0.008–2
CXM	I%	10.5	13.2	50.0	65.7	75.0	43.8
	R%	0	0	4.2	20.0	10.7	7.6
	MIC50(μg/ml)	0.023	0.032	1.5	2	2	1.5
	MIC90(μg/ml)	2	1.5	3	4	4	3
	MIC Range(μg/ml)	0.016–2	0.008–2	0.016–4	0.032–4	0.25–6	0.008–6
CRO	I%	0	0	4.2	17.1	10.7	6.9
	R%	0	0	0	0	7.1	1.4
	MIC50(μg/ml)	0.016	0.032	0.5	0.75	0.5	0.38
	MIC90(μg/ml)	0.38	0.38	1	1.5	2	1
	MIC Range(μg/ml)	0.004–0.5	0.008–0.75	0.016–1.5	0.008–2	0.25–8	0.004–8
IPM	I%	10.5	18.4	50.0	62.9	100	49.3
	MIC50(μg/ml)	0.012	0.032	0.094	0.125	0.19	0.094
	MIC90(μg/ml)	0.125	0.125	0.19	0.19	0.25	0.19
	MIC Range(μg/ml)	0.004–0.125	0.004–0.19	0.012–0.19	0.012–0.19	0.125–0.38	0.004–0.38
TCY	I%	0	5.3	8.3	40.0	82.1	28.5
	R%	94.7	86.8	75.0	28.6	10.7	56.9
SXT	I%	0	0	0	0	0	0
	R%	84.2	86.8	45.8	20.0	3.6	47.2
CHL	R%	52.6	18.4	20.8	0	0	15.3

*PEN* penicillin, *AMC* amoxicillin–clavulanic acid, *CXM* cefuroxime, *CRO* ceftriaxone, *IPM* imipenem, *TCY* tetracycline, *CHL* chloramphenicol, *SXT* trimethoprim–sulfamethoxazole, - no data for disk diffusion test, *I* intermediate, *R* resistant, *MIC50* minimum inhibitory concentration at which 50 % of the strains were inhibited, *MIC90* minimum inhibitory concentration at which 90 % of the strains were inhibited, *MIC range* range of minimum inhibitory concentration

### Macrolide resistance genes

The *ermB* gene was detected in all of the 143 erythromycin resistant isolates, and the *mefA* gene was detected additionally in 13 (9.1 %) isolates.

### DHFR amino acid sequences

The DHFR sequences of the 144 strains were compared with that of *S. pneumonia* ATCC 496 l9 (EMBL Z74778). The DHFR coding sequence, consisting of 504 nucleotides, encoding 168 amino acids, in trimethoprim-susceptible isolates showed a divergence of 3 to 7 nucleotides (0.6 to 1.4 %) compared with the ATCC 49619 isolate, resulting in 2 to 4 variations in amino acid sequence (1.2 to 2.4 %). DHFR coding sequences of trimethoprim-resistant isolates showed a 37- to 38-nucleotide divergence (7.3 to 7.5 %) from *S. pneumonia* ATCC 496 l9, resulting in 2 to 30 differences (1.2 to 17.9 %) in amino acid sequence (Table 3). In the present data, Ile100-Leu variation was determined in all of the trimethoprim-resistant isolates and none of the trimethoprim-susceptible isolate. Frequent variation in trimethoprim-resistant isolates with no change in trimethoprim-susceptible isolates included Glu20-Asp (97.1 % resistant isolates), Glu94-Asp (83.8 % resistant isolates), Leu135-Phe (97.1 % resistant isolates).

**Table 3:** The amino acid mutation of dihydrofolate reductase of trimethoprim-sulfamethoxazole-susceptible and –resistant isolates

Reference sequence^a^		Sequence types of SXT-susceptible isolates			Sequence types of SXT-resistant isolates																
sites	Amino acid	S1 (6)	S2 (15)	S3 (55)	R1 (1)	R2 (1)	R3 (1)	R4 (1)	R5 (1)	R6 (1)	R7 (1)	R8 (1)	R9 (2)	R10 (2)	R11 (3)	R12 (4)	R13 (5)	R14 (6)	R15 (11)	R16 (12)	R17 (15)
1	Met	-	-	-	-	-	-	-	-	-	-	-	-	-	-	-	-	-	Ile	-	-
6	Val	-	-	-	-	-	-	-	-	-	-	Ile	-	-	-	-	-	-	Ile	-	-
13	Glu	-	-	Lys	-	-	-	-	-	-	-	-	-	-	-	-	-	-	-	-	-
14	Glu	-	-	-	-	-	-	-	-	-	-	-	-	-	-	-	-	-	-	Asp	-
20	Glu	-	-	-	-	Asp	Asp	Asp	Asp	Asp	-	Asp	Asp	Asp	Asp	Asp	Asp	Asp	Asp	Asp	Asp
26	His	-	-	-	-	Tyr	-	-	Tyr	-	-	-	-	-	-	Tyr	Tyr	-	Tyr	-	Tyr
32	Gln	-	-	-	-	-	-	-	-	Lys	-	-	-	-	-	-	-	-	Arg	-	-
53	Met	-	-	-	-	-	-	-	-	-	-	-	-	-	-	-	-	Ile	-	-	-
60	Lys	-	-	-	-	-	-	-	-	Gly	-	Gln	-	-	-	-	-	-	-	-	-
70	Pro	-	-	-	-	Leu	Ser	-	-	Val	Ser	Leu	Leu	Ser	Ser	Ser	Ser	Ser	Ser	Ser	Arg
73	Lys	-	-	-	-	-	-	-	-	Ser	-	-	-	-	-	-	-	-	-	-	-
74	Ile	-	-	-	-	-	-	-	-	-	-	-	-	-	-	-	-	-	-	Leu	-
75	Asp	-	-	-	-	-	-	-	-	Glu	-	-	-	-	-	-	-	-	-	-	-
78	Ala	-	-	-	-	Thr	Thr	Thr	Thr	Leu	-	-	-	-	-	Thr	-	-	Thr	-	Thr
79	Thr	-	-	-	-	-	-	-	-	Val	-	-	-	-	-	-	-	-	-	-	-
81	Gln	-	-	-	-	-	Tyr	Tyr	Tyr	-	Tyr	His	His	Tyr	His	Tyr	His	His	Tyr	His	Tyr
82	Asp	-	-	-	-	-	-	-	-	Asn	-	-	-	-	-	-	-	-	-	-	-
83	Val	-	-	-	-	Ile	-	-	-	-	-	-	-	-	Ile	-	Ile	-	-	-	-
84	Gln	-	-	-	-	-	-	-	-	Glu	-	-	-	-	-	-	-	-	-	-	-
85	Ser	-	-	-	-	-	-	-	-	Asp	-	-	-	-	-	-	-	-	-	-	-
88	Asp	-	-	-	-	-	-	Gly	-	-	-	-	-	-	-	-	-	-	-	-	-
91	Gln	-	-	-	-	-	-	-	-	His	-	-	-	-	-	His	-	-	-	His	-
92	Asp	Ala	Ala	Ala	-	Gly	Ala	Ala	Ala	Asn	Ala	Ala	Ala	Ala	Ala	Ala	Ala	Val	Ala	Ala	Ala
94	Glu	-	-	-	-	Asp	Asp	-	Asp	Asp	Asp	-	-	Asp	Asp	Asp	Asp	-	Asp	Asp	Asp
99	Ile	-	-	-	-	-	-	-	-	-	-	-	-	-	Val	-	-	-	-	-	-
100	Ile	-	-	-	Leu	Leu	Leu	Leu	Leu	Leu	Leu	Leu	Leu	Leu	Leu	Leu	Leu	Leu	Leu	Leu	Leu
105	Ile	-	-	-	-	-	-	-	-	-	-	-	-	-	-	Val	-	-	-	-	-
108	Ala	-	-	-	-	-	-	-	-	Leu	-	-	-	-	-	-	-	-	-	-	-
110	Glu	Gly	-	-	-	-	-	-	-	-	-	-	-	-	-	-	-	-	-	-	-
111	Pro	-	Ala	Ala	-	-	Ser	-	-	-	-	Ser	-	-	-	-	-	Ser	Ser	-	Ser
116	Val	-	-	-	-	-	-	-	-	Ile	-	-	-	-	-	-	-	-	-	-	-
120	His	-	-	-	-	Gln	-	Gln	-	Gln	-	-	-	-	-	Gln	-	-	Gln	-	Gln
124	Arg	-	-	-	-	-	-	-	-	Gln	-	-	-	-	-	-	-	-	-	-	Lys
130	Tyr	-	-	-	-	-	-	-	-	Phe	-	-	-	-	-	-	-	-	-	-	-
133	Glu	-	-	Gly	Lys	-	-	-	-	-	-	-	-	-	-	-	-	-	-	-	-
134	Glu	-	-	-	-	-	-	-	-	Asp	-	-	-	-	-	-	-	-	-	-	-
135	Leu	-	-	-	-	-	Phe	Phe	Phe	Phe	Phe	Phe	Phe	Phe	Phe	Phe	Phe	Phe	Phe	Phe	Phe
137	Leu	-	-	-	-	-	-	-	-	-	-	-	-	-	-	-	-	-	S	-	-
138	Ser	-	-	-	-	-	-	-	-	Thr	-	-	-	-	-	-	-	-	-	-	-
139	Leu	-	-	-	-	Ile	-	-	Ile	Val	Ile	-	-	Ile	-	-	-	-	-	-	-
142	Thr	-	-	-	-	-	-	-	-	-	-	-	-	-	-	-	-	Ile	Met	-	Pro
144	Ser	-	-	-	-	-	-	-	-	Ala	-	-	-	-	-	-	-	-	-	-	-
147	Phe	-	-	-	-	-	-	Ser	Ser	Ser	Ser	Ser	Ser	Ser	-	Ser	Ser	Ser	-	Ser	Ser
148	Tyr	-	-	-	-	-	-	-	-	-	-	-	-	-	-	Phe	-	-	-	-	-
149	Ala	-	-	-	-	-	-	Thr	Thr	Thr	Thr	Thr	Thr	Thr	Val	Thr	Thr	Thr	-	Thr	Thr
150	Lys	-	-	-	-	-	-	-	-	Arg	-	-	-	-	-	-	-	-	-	-	-
155	Pro	-	-	-	-	-	-	-	-	Ala	-	-	-	-	-	-	-	-	-	-	-
160	Ile	-	-	-	-	-	-	-	-	-	Thr	-	-	-	-	-	-	-	-	-	-
161	Gln	-	-	-	-	-	-	-	-	Glu	-	-	-	-	-	-	-	-	-	-	-
162	Tyr	-	-	-	-	-	Cys	-	-	-	-	-	-	-	-	-	-	-	-	-	-

*S* SXT-susceptible isolates, *R* SXT-resistant isolates, - The same with the ATCC49619

^a^The amino acid sequence of ATCC49619 (EMBL Z74778)

### MLST

**Fig. 1:**
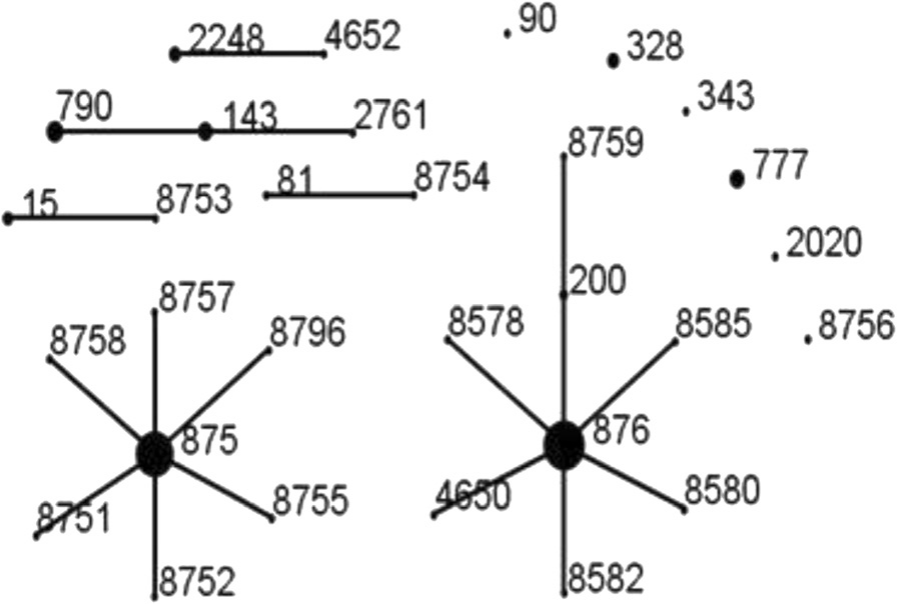
Population snapshot of 144 *S. pneumonia* strains revealed by an eBURST analysis. Lines indicate the presence of single-locus-variant links in particular sequence types, indicated by circles. The size of the circle corresponds to the number of isolates belonging to the sequence type (ST)

MLST analysis revealed 30 STs, the most common of which were ST876 (49/144, 34.0 %), ST875 (41/144, 28.5 %), ST790 (8/144, 5.6 %), ST143 (6/144, 4.2 %) and ST777 (6/144, 4.2 %). eBURST analysis revealed the presence of six CCs and six singletons (Fig. 1). CC876 was the most common group comprising 8 STs and accounted for 39.6 % of all strains while CC875 accounted for 32.6 % of the studied strains.

**Fig. 2:**
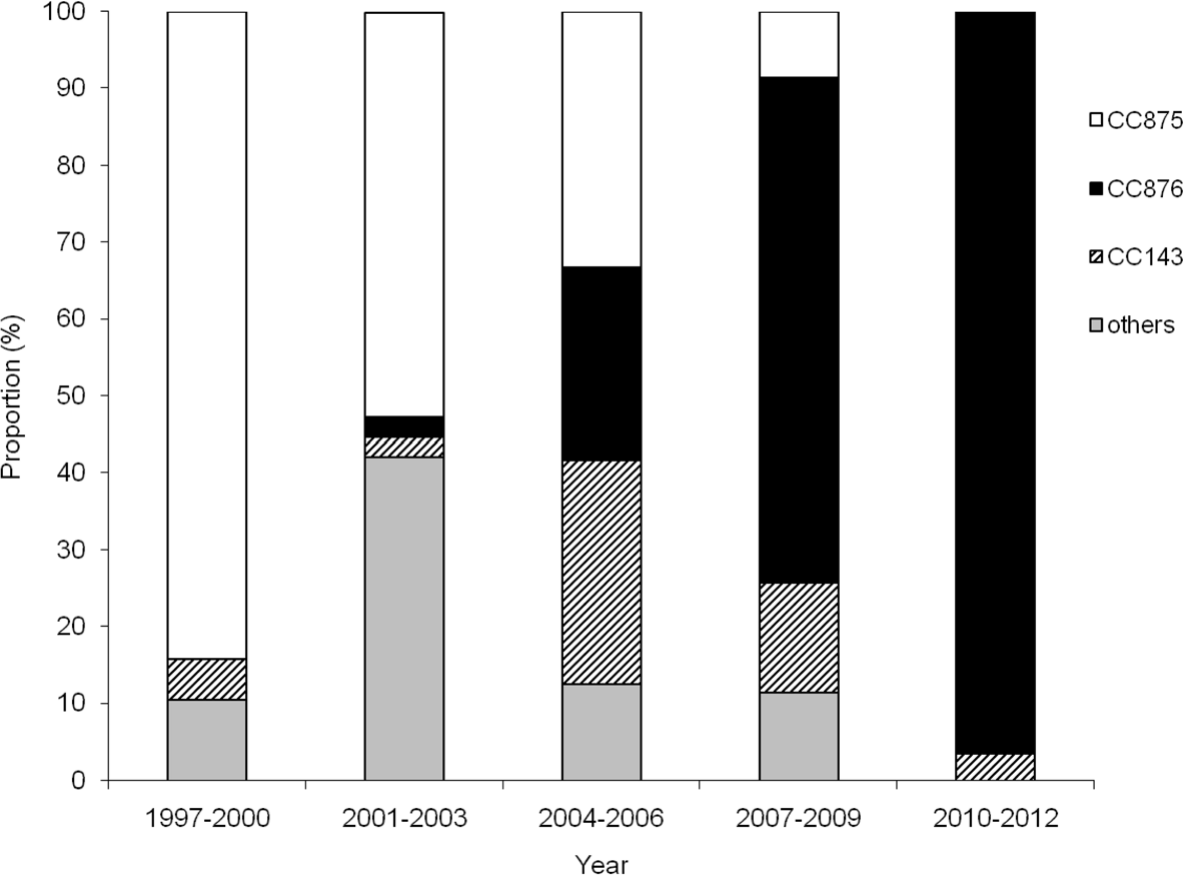
Distribution of the common CCs of serotype 14 *S. pneumonia* isolates during the study period

Figure 2 shows the distribution of the common CCs in serotype 14 *S. pneumonia* strains during the study period. From 1997 to 2012, the frequency of CC876 increased from 0 % in 1997–2000 to 25 % in 2004–2006, and reached 96.4 % in 2010–2012, whereas CC875 decreased from 84.2 to 33.3 % and finally to 0 % in the corresponding periods.

**Fig. 3:**
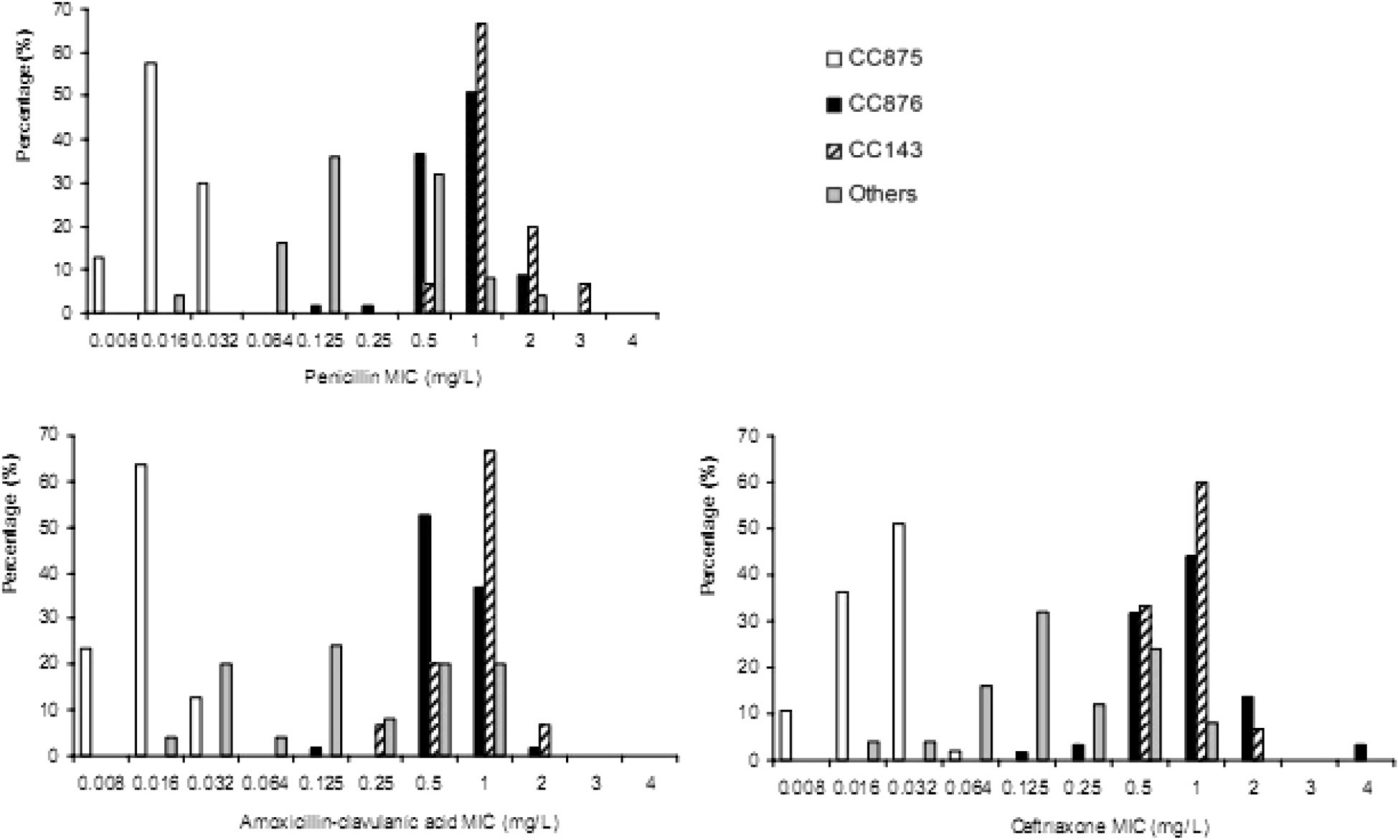
MIC distribution of the common CCs of serotype 14 *S. pneumonia* isolates to β-lactam antibiotics

Significant differences in β-lactam antibiotic resistance between the predominant CCs were found in this study (Table 4). All CC875 strains were susceptible to β-lactam antibiotics while CC876 and CC143 isolates showed high β-lactam antibiotic non-susceptibility and MICs (Fig. 3). By contrast, CC875 were significantly more resistant to trimethoprim-sulfamethoxazole and chloramphenicol than CC876. Both clones exhibited similar resistance to erythromycin and tetracycline.

**Table 4:** Difference between the antimicrobial susceptible profiles of CC876 and CC875 of serotype 14 *S. pneumonia*

Antibiotics	Susceptibility and MIC	CC876 (*n* = 57)	CC875 (*n* = 47)	CC143 (*n* = 15)	Others (*n* = 25)	Total
PEN	I%	0	0	6.7	0	0.7
	MIC50(μg/mL)	0.75	0.016	1	0.125	0.5
	MIC90(μg/mL)	1	0.023	1.5	0.75	1
	MIC Range(μg/ml)	0.125–2	0.004–0.032	0.38–3	0.016–1.5	0.004–3
AMC	MIC50(μg/mL)	0.5	0.016	1	0.125	0.38
	MIC90(μg/mL)	1	0.023	1	1	1
	MIC Range(μg/ml)	0.094–1.5	0.008–0.023	0.25–2	0.016–1	0.008–2
CXM	I%	52.6	0	66.7	36	43.8
	R%	36.8	0	26.7	0	7.6
	MIC50(μg/mL)	2	0.023	2	0.5	1.5
	MIC90(μg/mL)	4	0.032	3	2	3
	MIC Range(μg/ml)	0.19–6	0.008–0.125	1–4	0.016–2	0.008–6
CRO	I%	15.8	0	6.7	0	6.9
	R%	3.5	0	0	0	1.4
	MIC50(μg/mL)	0.75	0.023	0.75	0.125	0.38
	MIC90(μg/mL)	1.5	0.032	1	0.5	1
	MIC Range(μg/ml)	0.125–8	0.004–0.047	0.38–1.5	0.016–1	0.004–8
IPM	I%	43.9	0	33.3	4	49.3
	MIC50(μg/mL)	0.125	0.016	0.125	0.064	0.094
	MIC90(μg/mL)	0.19	0.032	0.19	0.125	0.19
	MIC Range(μg/ml)	0.047–0.38	0.004–0.064	0.064–0.19	0.012–0.19	0.004–0.38
TCY	I%	68.4	0	6.7	4	28.5
	R%	5.3	95.7	93.3	80	56.9
SXT	I%	0	0	0	0	0
	R%	3.5	91.5	0	92	47.2
CHL	R%	1.8	31.9	13.3	16	15.3

*PEN* penicillin, *AMC* amoxicillin–clavulanic acid, *CXM* cefuroxime, *CRO* ceftriaxone, *IPM* imipenem, *TCY* tetracycline, *CHL* chloramphenicol, *SXT* trimethoprim-sulfamethoxazole, -, no data for disk diffusion test, *I* intermediate, *R* resistant, *MIC50* minimum inhibitory concentration at which 50 % of the strains were inhibited, *MIC90* minimum inhibitory concentration at which 90 % of the strains were inhibited, *MIC range* range of minimum inhibitory concentration

## Discussion

The present study showed that serotype 14 isolates was not very common in all of the *S. pneumonia* (7.3 %) strains isolated from Chinese children. But its percentage in the whole *S. pneumonia* isolates over time increased from 3.2 % in 1997–2000 to 13.8 % in 2010–2012. Epidemiology data in a previous report showed that the percentage of serotype 14 isolates was usually lower than 10.0 % [21]. From January 2005 to December 2006, a total of 451 *S. pneumonia* isolates were collected from 9 study centers in 8 cities across China, of which the most prevalent serotypes in descending order were 19 F (42.1 %), 19A (11.8 %), 14 (7.5 %), 6B (7.3 %), 23 F (5.8 %), and 15 (4.4 %) [22]. Our previous study also revealed that among 338 pneumococcal isolates, the most common were 19 F (55.6 %), 19A (13.9 %), 23 F (10.1 %), 6B (4.7 %), and 14 (3.6 %) [23]. But among the isolates causing invasive pneumococcal disease (IPD) in children, serotype 14 may play a more significant role. In a study [14] conducted from 2006 to 2008 in 11 hospitals across China, 171 *S. pneumonia* isolates collected from children suffering from IPD under the age of 14 were analyzed for serotype distribution, and the most prevalent serotype was 19 F (19.9 %), followed by serotype 14 (19.3 %). In another previous study in China [12], a total of 61 invasive strains were isolated in Shenyang, the serotype distribution of those isolates were 19A (41.0 %), 14 (19.7 %), 19 F (11.5 %) and 23 F (9.8 %). Data from developed countries before the introduction of the conjugated vaccines indicated that the proportion of serotype 14 was 15.7 to 19.8 % [8, 24]. In September 2008, the 7-valent pneumococcal conjugated vaccine (PCV7) became available for the private sector in China. However, the vaccination rate is very low (less than 1.0 %). Thus, the frequency of serotype 14 did not decrease in 2012, and even increased. During the investigation period, the susceptibility to beta-lactam antibiotics of serotype 14 isolates decreased. We also found a significant clonal shift of serotype 14 strains from CC875 to CC876 over time. Given the discrepancy of antibiotic resistance between these two CCs, the genotype replacement was deduced to be caused by the antibiotic selective pressure. The spread of the highly resistant CC876 could be driven by the selective pressure from antibiotic use which limited the spread of the generally susceptible CC875 at the same time. Previous study suggested that the β-lactam antibiotics, especially the cephalosporin were frequently used in pediatric clinic work [25]. Chloramphenicol has not been used medically since the 1990s, hence, the resistance rate against Chloramphenicol decreased. Our previous study on serotype 19 F [9] and 23 F [10] *S. pneumonia* isolates indicated that the wide use of beta-lactam antibiotics not only selected the resistant *S. pneumonia*, but also other epidemiological characteristics, such as serotypes and genotypes. The suggestion is corroborative by the present results.

The genotype replacement of serotype 19 F and 23 F *S. pneumonia* isolates in China obviously resulted from the input of highly resistant international pneumococcal clones (CC271 and ST81) [9, 10]. Little was known about the spread of serotype 14 *S. pneumonia*. There was no sufficient evidence to support that it was associated with some international spread CCs of serotype 14 strains. There were 1594 serotype 14 *S. pneumonia* isolates in the MLST database (http://spneumoniae.mlst.net/) from the whole world. The eBurst analysis of these data showed that the common CCs were as follows: CC15 (32.3 %), CC156 (17.8 % including ST143), CC63 (12.1 %), CC124 (10.7 %) and CC230 (5.7 %). The predominant CC875 and CC876 in the present study covered only 30 (1.9 %) and 13 (0.8 %) strains in the MLST database, respectively. The PMEN notes international spread resistant clones of serotype 14 including ST18, ST9, ST20, ST67, ST230 and ST124, but they were rare in our data.

It was noted that the resistance rate of serotype 14 *S. pneumonia* isolates to trimethoprim-sulfamethoxazole decreased significantly during the study period. CC876 and CC143 strains were susceptible to trimethoprim-sulfamethoxazole. We did not find the similar results in serotype 19 F [9] and 23 F [10]. The DHFR genes were amplified and sequenced, which confirmed the phenotype test. It was reported [26] that trimethoprim resistance is an essential component of co-trimoxazole resistance. Very few (6 %) of the trimethoprim-resistant isolates were sensitive to co-trimoxazole. A previous study [27] demonstrated that the mutation of Ile100-Leu was critical to the generation of trimethoprim resistance. The results presented here were consistent with the requirement for this mutation but also demonstrated that other changes [28] had considerable impacts on the function of the enzyme. Overall levels of nucleotide divergence from the reference strain were similar in the study, at between 6.0 and 7.5 % for resistant isolates and under 1.5 % for susceptible isolates. The significant epidemiological character should be confirmed by more data.

The study has a limitation. The survey period was divided into five non-equal study period for enough quantity isolates in statistic analysis. Considering the number (144 isolates) of the isolates in this study, we believe that the present result demonstrated the epidemiological changes and molecular characteristics of serotype 14 *S. pneumonia* in China objectively.

## Conclusions

In summary, we found an increase in the prevalence of serotype 14 *S. pneumonia* isolates with increasing β-lactam antibiotic resistance in China from 1997 to 2012. It was indicated that CC876 replaced CC875 under the pressure of antibiotics. Further long-term surveys of serotype 14 *S. pneumonia* are required to monitor the prevalent STs and antibiotic resistance of this important human pathogen.

## Abbreviations

PCR: Polymerase chain reaction
ST: Sequence type
MLST: Multilocus sequence typing
CC: Clonal complex
MDR: Multidrug resistant
MIC: Minimum inhibitory concentration
DHFR: Dihydrofolate reductase
PCV7: The 7-valent pneumococcal conjugated vaccine
IPD: Invasive pneumococcal disease
